# Solidarity in the Time of COVID-19?

**DOI:** 10.1017/S0963180120000791

**Published:** 2020-10-09

**Authors:** FLORIS TOMASINI

**Keywords:** COVID-19, pandemic, solidarity, anthropocentric (solidarity), biocentric (solidarity), utopia, heterotopia

## Abstract

This article critically examines how solidarity has been enacted in the first 2 months of the COVID-19 pandemic, mainly, but not exclusively, from a United Kingdom perspective.^1^ Solidaristic strategies are framed in two ways: aspirations to overcome COVID-19 (utopian anthropocentric solidarity); and those that are illusory, incompatible, contradictory, and disrupting of solidaristic ideals (heterotopian solidarity). Solidarity can also be understood more widely from a biocentric perspective (solidarity with all life). In the context of COVID-19 a lack of biocentric solidarity points to a probable cause of the pandemic; where COVID-19, harmless in bats, jumped species as a consequence of closer contact with humans. Solidarity, therefore, is not only expressed in a fight against a viral “enemy” but is also a reminder of human activity that has upset balances within ecosystems.

## Introduction

COVID-19 emerged in Wuhan in late 2019, a city in China’s Hubei province with a population of 11 million people. COVID-19 grew by several thousand per day in China in late January and early February 2020, the peak of the epidemic in China.^2^ COVID-19 spread east and was well established in Europe by mid-March. Within 5 months it had affected almost every country in the world.

One striking feature of this pandemic is how it bound people together to stand in solidarity against COVID-19, with the common purpose of slowing infectivity, morbidity, fatality from the disease, as well as to ameliorate indirect harms of coronavirus to our taken for granted way of life.

People from different countries have adopted all kinds of solidaristic strategies in response to the pandemic at various levels of social order. It is beyond the scope of this paper to make any grand claims for this work – either as a robust national or international comparative study. It is possible, however, to critically *explore* the early stages of the pandemic through the lens of solidarity in the United Kingdom and beyond.

## Understanding Solidarity

Solidarity is a form of agreement, especially among individuals with a common interest. The meaning of solidarity is also associated with an “awareness of shared interest, objectives, standards and sympathies creating some sense of groups or classes.”^3^

The sociologist and bioethicist Barbara Prainsack and Alena Buyx have critically examined solidarity in *“*Solidarity in Biomedicine and Beyond.*”* ^4^ Solidarity, they observe, entered political consciousness in the heat of the French revolution with the rallying cry of *fraternité*. They go on to tease out some of the essential elements of solidarity including a sense of “being bound together” through, for instance, “sharing similar objectives or circumstances; mutual assistance and help, particularly in situations of hardships; symmetric relationships between those engaged in solidarity” (i.e., despite other parts of their lives not being equal or even similar); and “a link to individual and collective well-being.”^5^ They elaborate on the idea of solidarity as “enacted commitments to accept costs (financial, social, emotional or otherwise) to assist others with whom a person or persons recognise similarity in a relevant respect.”^6^ Interestingly “persons who recognise similarity in a relevant respect,” in Prainsack and Buyx terms, are “bound together in solidarity as opposed to charity.” This is because solidarity is primarily affirming our shared similarity, whereas charity is making up for perceived differences in human needs to be compensated for.

Solidarity, therefore, may be understood as the collective good of being bound together in order to provide mutual assistance through what we perceive as some form of common interest. There are four further conceptual framings that help illuminate solidarity. It is worth outlining these framings briefly before interpreting them further within the context of COVID-19.

1. Human solidarity can be broadly conceptualized in respect to two ideal types.
  One ideal type of human solidarity is purely motivated by shared sympathy and fellow-feeling. Epicurean friendship can be understood in this way, where the solidarity of brotherly love (*philia*), in classical times, was understood as a high ideal and a good in itself.^7^
  Another ideal type is born out of necessity and is characterized by solidarity wrought out of hardship and a *just* struggle against what is perceived as a legitimate foe or enemy.
  Solidarity in the face of COVID-19 is born out of an aspirational struggle and enmity that defined it. Put more fulsomely, any fellow-feeling that brings much of the world together in solidarity against COVID-19 arises out of an aspirational struggle with an “invisible killer” that threatens to extinguish our very existence and threaten a way of life.
2. One can break down solidarity into levels of social order.
  Solidarity can be understood at different levels of social ordering, from interpersonal relations, customs, and habits of groups, to larger social structures and institutions of relating and behaving. Prainsack and Buyx have recognized this, and have identified three tiers of solidarity. Tier 1, interpersonal solidarity, manifests “as an individual willing to carry the costs to assist others.” Tier 2 involves group solidarity “where there is a collective or shared commitment to carry costs to assist others.” Tier 3 is considered the institutional formalization of solidarity where solidarity becomes entangled in “contractual, legal or administrative norms of behaviour.”^8^ These solidaristic tiers of social ordering map onto examples of solidarity in the time of COVID-19.
3. Human-centered solidarity is an interplay between its aspirational ideals and its real enacted effects. In other words, we also need to interpret solidarity in the context of COVID-19 as an alternate social ordering (heterotopia) that contradicts its aspirational ideals.
4. Solidarity can be interpreted narrowly, in human-centered, and/or more broadly in biocentric terms.
  It is taken for granted that coronavirus is the enemy and that human beings should bind together in solidarity to overcome it in order to return to business-as-usual. Although we do need to stand in solidarity against a virus that is harming and killing us, it is by no means necessary that we need to restore the socioeconomic systems that may have unleashed it in the first place. We may need to reevaluate solidarity, from a wholly human-centered concept to one that is in solidarity with all life (biocentric solidarity).

## Anthropocentric Solidarity in the Context of COVID-19: A Utopian Perspective

Because COVID-19 directly threatens human life, as well as indirectly harms our way of life, it has been deemed “an enemy.” This is clear in much of the rhetoric around solidarity. Prominent figures, like the director general of WHO, Dr Tedros Adhanon Ghebreyesus; the French president, Emannuel Macron; and the British prime minister Boris Johnson have all mobilized martial metaphors:

On the 12 March 2020 Tedros Adhanon Ghebreyesus called for social solidarity from global institutions and governments to prevent the spread of the virus:

“This outbreak is a test of solidarity – political, financial, and scientific. We need to come together to fight a ‘common enemy’ [my inverted commas].”^9^

On the 16 March Emmanuel Macron ordered the French people to stay at home, anticipating the closing of the country’s land-borders declaring that:

“…we are at war with ‘an invisible enemy,’ and the measures were unprecedented, but circumstances demanded them [my inverted commas].”^10^

Boris Johnson similarly described the conflict with the virus, through a range of martial metaphors. In March he described the crisis as “the fight…in which every one of us is directly enlisted.”^11^

Utopia comes from the Greek which translates as “no-place” (literally a nonexistent society). Thomas More pointed out the similarity between *utopia* and *eu-topia*—which is from the Greek “good” or “well” place.^12^ Hence utopia, in modern English can also mean “good place” as well as a nonexistent place.

Utopia as a good place can be further articulated in terms of a tiered hierarchy of social ordering that promotes human solidarity as an aspiration in the context of the COVID-19 pandemic.

The first major solidaristic aspiration in the fight with “the invisible enemy” was to slow the viral contagion. The United Kingdom government policy was deeply influenced by negative public perception and a scientific modeling paper published by an Imperial College London team led by Prof. Neil Ferguson which estimated 40 million deaths globally if no measures at all were taken (which could only be halved if mitigation strategies were pursued).^13^

Mitigation involved shielding the most vulnerable in society at home, or in care homes, whilst adopting a herd immunity policy for the rest. Herd immunity would slow the transmission of the virus without artificially interrupting it through social distancing and a lock-down. The idea behind herd immunity was to allow a significant percentage of the population to be infected in order to provide natural protection for the rest. The problem with a mitigation strategy, from the perspective of Scientific Advisory Group for Emergencies (SAGE) and Downing Street, was that there was a paucity of information about the virus and how it would affect the National Health Service (NHS), and its capacity to cope with most vulnerable COVID patients. Mitigation was seen as a potential public health disaster that would be perceived as callous by the UK public.

A suppression strategy was seen as the way forward where according to the Imperial College modelers:

“healthcare demand can only be kept within manageable levels through the rapid adoption of public healthcare measures (including testing and isolation of cases and wider social distancing measures) to suppress transmission, similar to those being adopted in many countries at the current time.”^14^

This led the United Kingdom to switch from herd immunity to lock-down (a wider social distancing measure). This finally took effect in the United Kingdom on 23 March 2020 and was in line with other EU countries who acted earlier.

Lock-down, in the context of the United Kingdom, is a form of government guidance, directing the national population to stay at home and only go out for essential supplies like food and medicine and for a brief period of exercise between 30 to 60 minutes.^15^ Whilst this was made much less strictly enforceable in the United Kingdom than in some other EU countries, the idea behind lock-down was to implement enforceable solidaristic regulations with voluntary cooperation of the public.

In more technical terms it was a way of encouraging interpersonal solidarity (Tier 1) with solidarity that could be legally enforced through a lock-down (Tier 3). In other words, a vital precondition for solidaristic laws and regulations to be effective was the voluntary acceptance of them by the public.^16^ It was believed that draconian policies to limit personal freedoms in a lock-down (Tier 3) was only acceptable, in a fairly libertarian state like the United Kingdom, if people at the interpersonal level stood in solidarity with it (Tier 1).

The stick and carrot of solidarity in an emergency pandemic relied on clear messaging, always keeping the public onside with Government policy led by ‘the science.’ This was captured in the government’s clearest message to the public on regular TV broadcasts that informed watchers about coronavirus strategy on a daily basis:

“Stay home. Protect the NHS. Save lives.”^17^

A much more spontaneous form of interpersonal solidarity (Tier 1) arose through clapping for the NHS. At 8 pm on Thursday nights, people throughout the nation stood outside their homes to show their appreciation for frontline NHS workers by clapping and banging saucepans. Started by a Dutch ex-patriate Annemarie Plas living in London, this lasted for just over 2 months (from 26 March to 28 May 2020) and had been originally inspired by a similar event in the Netherlands.^18^

Clapping for the NHS not only boosted morale and showed appreciation for frontline NHS workers, it helped keep group solidarity (Tier 2), where there was a shared commitment to carry the cost for others by staying at home, protecting the NHS and saving lives. In some ways, therefore, it was a movement that created a virtuous circle, especially in the darker days when transmission, hospitalization, and fatality were at its peak.

What was particularly striking is that libertarian governments, on the whole, had the support of their citizens to curtail freedoms more profoundly than any wartime leader. The spread of the virus also prompted an unprecedented solidarity to save our socioeconomic life: normally fiscally prudent Governments approved billions in socioeconomic relief schemes to support citizens in times of economic hardship, as well as boosting healthcare and emergency spending to tackle the virus outbreak.

In March through to April 2020 alone, the UK chancellor of the exchequer announced an unprecedented government-backed fund to support employees and businesses. Solidarity, at Tier 3, to support the economy was primarily designed to future-proof any group solidarity (Tier 2) that might disappear if people had no employment or businesses to return to post-pandemic.

On 17 March, the initial investment was calculated at £330 billion of guarantees (the equivalent to 15 percent of GDP). This included all kinds of support to the public and business, most notable the new Coronavirus Job Retention Scheme on 20 March. This scheme enabled almost any business whose operations have been severely affected by the current COVID-19 pandemic to furlough employees and apply for a grant that covers up to the lower of 80 percent of an employee’s regular wage.^19^

Solidarity (at Tier 3) was not only national, it also had a supranational in character. This is nicely illustrated by both Europe and the WHO.

If the United States has been protectionist about how to handle the pandemic, withdrawing funding from WHO and blaming the Chinese for what Trump called “the Wuhan virus,” the EU have taken solidaristic multilateralism further in response to COVID-19 within the European bloc.

As Josef Borrell puts it: “only by pulling together and cooperating across borders can we beat the virus and contain its consequences—and the EU has a central role to play.”^20^ This has played out practically through raising joint procurement orders for Personal Protective Equipment (PPE) for health workers across the EU; in the treatment of French and Italian patients in German hospitals; in the shipment of medical equipment to Italy; in the support of EU citizens returning home; and, in the European Central bank approving 750 billion euros to do “everything necessary” to deal with the crisis.^21^

Given Ghebreyesus’s call for solidarity, it is unsurprising that the WHO and partners have launched a clinical trial for COVID-19 with the name “Solidarity.” The idea behind the solidarity trial is to enrol patients in multiple countries to rapidly discover whether any drugs slow disease progression or improve survival. This trans-national effort is remarkable given that before the pandemic, regimes of power normally encouraged competition in clinical innovation. The upside of such trials, before the pandemic, was to refine them to high specificity, rigor, and standard to warrant quality with a price tag. The downside was that clinical innovation was often profit-led and not highly affordable.^22^

The WHO clinical solidarity trial has tried to mitigate this simplifying procedure “to enable even overloaded hospitals (with no paperwork) to participate across 100 countries.” The idea behind this initiative being to “find safe and effective therapeutics as soon as possible, whilst also encouraging developers and companies to collaborate to ensure effectiveness and affordability.”^23^

Although solidarity is maintained through national and supranational levels, up to Tier 3, it also involves interpersonal solidarity and community solidarity at Tier 1 and 2.

There are a number of examples.

Tier 1 and Tier 2 solidarity is entangled, and can be illustrated through two clear examples. First, clapping for the NHS in the United Kingdom binds individuals together in a spirit of solidarity with their communities and local hospitals. Second, solidarity at an interpersonal level was expressed in the joy of group togetherness by quarantined Italians singing together from balconies in places like Sienna.^24^

Interpersonal solidarity is also entangled through the group by a rise in family and friend group cohesion through video-conferencing which has enabled people to talk to each other in times of lock-down;^25^ volunteer cottage industry to make masks, gowns, and PPE in the United Kingdom;^26^ neighborhood WhatsApp groups that are organized to help the vulnerable practically with groceries and prescriptions medicines.^27^ These grass-root initiatives of interpersonal solidarity (Tier 1) grew into a group solidarity where the collective is willing to carry the cost for others (Tier 2).

There are examples which attempt to build grass-root solidarity from the top-down as well as the bottom-up, from Tier 3 down to Tier 1 and 2.

In the United Kingdom this is exemplified by the NHS Volunteer Responders scheme which was an initiative set-up by the health secretary, Matt Hancock, to support the NHS and care sector during the COVID-19 outbreak.^28^ The Responders scheme put potential volunteers and vulnerable people who needed groceries, medicines, and conversation in contact with each other. The vulnerable were identified through medical databases and potential volunteers were encouraged to download the Goodsamapp and were given a code of conduct and instructions to help the vulnerable if they were contacted. In 4 days of opening, it received 750,000 volunteers, three times the original target.^29^

Having spent some time characterizing the nature of aspirational solidaristic strategies present during the COVID-19 emergency, it is necessary to also understand how the enacted reality often contradicted the aspiration.

## Anthropocentric Solidarity in the Context of COVID-19: A Heterotopian Perspective

The idea of heterotopia comes out of the philosopher Michel Foucault’s work and is a parallel and often contradictory space, containing undesirable bodies and states that throw into stark relief the utopian idea of the good or ideal. Foucault uses the idea of heterotopia in different ways: as spaces of crisis that hide what is out of sight; as alternative spaces/institutions that house individuals that exhibit deviant behavior; as spaces that juxtapose objects from different times and places; as spaces of ritual; and finally as spaces where reality is thrown up uncomfortably alongside a space of illusion.^30^ Kevin Hetherington takes Foucault’s idea further by talking about heterotopia as “spaces of alternate social ordering” and as an “interplay between utopia and heterotopia.”^31^

If utopias around human solidarity during the pandemic may be expressed as an aspirational, the good of being bound together, then COVID heterotopias may be expressed as real and disturbing social orderings; where human solidarity becomes contradictory, illusory, in crisis, and deviant from expected norms of behavior.

In the making of solidarity at this time we need to return to the interplay between the aspirational and the contradictory; the illusory and the real, the norm and the deviant, crisis and success. For the sake of clarity, here is a numbered list to illustrate COVID heterotopias.

1. The appeal for solidarity during COVID-19 is often expressed in aspirational terms through a global effort. If we drill down into the problem, then some spaces and social orders present enormous challenges that contradict simple global solutions

Tedros Adhanom Ghebreyesus frequently calls for solidarity and global institutions and governments to prevent the spread of the virus. However, the developing world presents indomitable challenges compared to the developed world, presenting contradictory realities where measures to prevent contagion are much more challenging.

Take one area alone: the UN-habitat report estimates that 238 million people in the region of Sub-Saharan Africa are living in informal settlements without adequate basic services and social amenities that would enable them to follow the prescribed COVID-19 transmission measures.^32^ With such glaring structural inequalities in developing countries, low standards of living, and healthcare provision such areas are, potentially at least, unequal crisis points for transmission and morbidity from COVID-19.

Structural inequalities that exacerbate the spread of coronavirus do not only exist along the developing/developed world fault line; they are also present in developed countries, like the United Kingdom, where inequality has become deeper over the last two to three decades. People living in the poorest areas in the United Kingdom (usually in areas of high-urban density) are dying of COVID-19 at twice the rate of those in the richest areas. Statistically, there are 55.1 percent death per 100,000 in 10 percent of the most deprived places compared with 25.3 percent in the 10 percent least deprived.^33^

1. The appeal for solidarity in the COVID-19 pandemic is often made for all—regardless of economic, health, and genetic status. This, of course, presupposes that all people can expect the same treatment outcomes.

Although all people may be infected by coronavirus, not everybody that is infected will be symptomatic, and/or indeed be affected by COVID-19 in the same way. The danger of coronavirus is its infectivity rate (an R number 2–3 higher than seasonal flu), and pervasiveness. That is, whilst it is more lethal than flu, yet less lethal than SARS, if contracted, COVID-19 has killed five times as many people as SARS in 3 months.^34^

Solidarity, in terms of expecting treatment COVID-19 outcomes to be the same for everybody, is illusory. The success of treatment outcomes is not uniform amongst anyone who can potentially be infected. COVID-19 is least harmful in children and healthy people in their 20s and 30s; has a greater morbidity and fatality rate in older people, especially 60 plus; in middle-aged men rather than women; in people with certain underlying health conditions (especially heart and lung disease, compromised immune systems, diabetes, and people who are obese). In particular, it has proven to affect particular ethnic groups disproportionately where those with Black and Minority Ethnic (BAME) backgrounds in the United Kingdom are overrepresented in the death toll by 27 percent.^35^

The test of solidarity is more insuperable in groups who are already *primarily* vulnerable to COVID-19: age, genetic susceptibility to long-COVID, underlying health conditions, and race are all factors.

Heterotopian social ordering is more troubling when it exposes an uncomfortable reality alongside the illusion of solidaristic strategies that have been highly aspirational during the pandemic. Indeed, in the time of COVID-19, criticism of the UKs handling of the pandemic has been most biting where it has failed to secure key solidaristic aspirations.

1. On the surface, solidarity in the United Kingdom against the virus could have been achieved through an effective shielding strategy of older people, either at home or in care homes. However, in reality, this opened up two controversial issues: intergenerational fairness and the capacity to effectively protect older people in care homes from developing COVID-19.

Whose freedoms we curtail needs to reflect morbidity and mortality risks. One can appeal for solidarity here: protecting and shielding those who pose the greater risk because of their age from COVID-19 is a gesture of intergenerational solidarity. This is no simple matter without knowing how COVID-19 effects each generation and for what reasons. Without further justification and good reason shielding people just because people are old is, arguably, discriminatory and ageist.

It is easier to make the case for intergenerational solidarity if it is clear why some older people are more susceptible to dying from COVID-19. In care homes, for example, there are significant numbers of older people with multiple comorbidities. They have been particularly susceptible to fatality from COVID-19. As such the obvious strategy in the United Kingdom (and elsewhere) was to shield residents in care homes from being infected by COVID-19 and unnecessarily dying.

This aspiration for a good solidarity strategy to limit contagion numbers for coronavirus in the United Kingdom went awry when the suppression policy to flatten the COVID-19 curve focused on hospitals and not care homes. This resulted in reluctance, and sometimes refusal to treat older patients in the hospital for COVID-19, and a desire to discharge them back into care homes to prepare for the expected peak in hospital admissions. Moreover, in retrospect, it turned out that many elderly patients that had been discharged from hospitals without a COVID negative test back into care homes seeded the transmission of COVID-19 into the care home sector. This was exacerbated further by inadequate testing for residents and staff in care home settings, and not having adequate standards in PPE.^36^

In summary, a space of illusion—care homes where older people should have been shielded from COVID-19—actually exposed the reality of more disturbing spaces—care home residents and staff dying of COVID-19 in unusually large numbers.

A similar heterotopian reality is exemplified by the politicization of clapping for the NHS. Contradictory social orderings sat together awkwardly. On the one hand, clapping for the NHS was well-intentioned solidarity that supported frontline carers. On the other, this public support side-lined an alternative picture: frontline healthcare workers without adequate PPE dying in disproportionate numbers. The mirror of the solidarity ideal, clapping for frontline NHS staff, concealed a more disturbing reality; that it was okay to routinely support a system that risked its heroes becoming dead heroes.

Clapping for the NHS is heterotopian in that it “creates worlds within worlds, mirroring yet upsetting what is outside.”^37^

1. The official UK government discourse around handling the pandemic is solidaristic in its public aims and proclaimed success. Yet it conceals disturbing realities that have contradicted the good of binding together to tackle coronavirus effectively. This makes government discourses around handing the pandemic effectively, a heterotopia reality, as well as the utopian aspiration that achieved some limited successes.

The attention on human solidarity to combat COVID-19 falls on some basic facts that are hidden in plain sight. Regardless of all caveats that have to be made in any rigorous international comparison of how different countries fared—that is, the size of population, urban and ethnic mix, average age of citizens, and how deaths are recorded—the UK official death toll for COVID-19 is the highest in Europe.^38^ It is a mistake, for statistically comparative reasons, to claim that UK's highest death toll from COVID-19 makes it the worst European performer in the handling of the pandemic. This said it is indubitably true to claim that the United Kingdom performed poorly and that it performed less well with the pandemic than most of the European bloc. Whilst it is unskilful to make EU comparisons, it is possible to throw some light as to why the United Kingdom performed poorly in its handling of the pandemic.

What follows is a short summary of some of the heterotopian social orderings that undermined and disturbed the solidaristic aspirations set to deal with pandemic.

1. Speed: Solidarity against the spread of the virus with high-infectivity rate like coronavirus had to be quick and decisive, using an effective strategy that stopped exponential contagion. The United Kingdom was slow compared to other EU countries to employ a decisive strategy to contain the spread of coronavirus in the population, leading to a delayed and higher than the expected peak in morbidity and mortality. It is likely that indecision between the 12 and 16 March—where Johnson’s Government took no decisive steps to stop the spread of the virus (i.e., social distancing)—and 16 March when social distancing measures finally lead to a national lock-down on 23 March, led to higher transmission rates that cost more lives that may have been forecast.^39^ Indeed, it has been claimed by Prof. Neil Ferguson that the death toll from coronavirus in the United Kingdom could at least have halved if the lockdown had happened a week earlier^40^
2. Testing: Any effective solidarity strategy needed community testing and track and trace in order to find hotspots of viral infectivity early in order to stop further spread of the virus in the general population. In South Korea, mass community testing was prolific and organized and showed 18 regions in the country accounted for 84 percent of the cases.^41^ The United Kingdom made the mistake of stopping community mass testing early (12 March) in favor of a nonspecific policy of lock-down. Furthermore, the United Kingdom was much less prepared for testing early on, than either South Korea or Germany where testing capacity was already in place and expertise was in place and mobilized relatively quickly and efficiently. It has failed to deliver a 'world-beating' test and track system, or indeed a system staffed by local experts that works well.
3. PPE: Solidarity is partly about protection from being infected by the coronavirus. This required having stocks of PPE that were of high standard and fit for purpose in protecting key workers from coronavirus. With massively increased global demand for PPE supply, this was a global problem. Despite this, the UK stymied itself in getting the required quality PPE in a number of ways. Just before the outbreak of the pandemic, it had sold a large PPE manufacturer to the United States.^42^ The United Kingdom did not have a coordinated response to the procurement of PPE from all available sources. It has not shown solidarity with Europe’s joint procurement order for PPE for reasons that remain opaque.^43^ It also showed little solidarity with Small to Medium Enterprises (SME) willing to supply the state with PPE until late on.
4. Coordination (decisionmaking): Solidarity is partly about joined-up decision-making. There was no clear and coordinated decisionmaking rationale across the UK regions and within England. There are a plethora of key social actors adding to difficulties in coordination: Cobra; Downing Street, and its Advisers; SAGE; the chief medical officer and scientific officer; the National Health England; and Public Health England, to name but some of the key actors.
5. Coordination (sourcing): solidarity is also partially about sourcing what is needed to fight the pandemic. Prof. Anthony Costello, the director of the Institute of Global Health at the University College London has reported that the UK’s chaotic response “reflected the wholesale destruction of a co-ordinated and focused state sector. Outsourcing, delegated powers, internal markets… have made a single response impossible. It affects every aspect of policy.”^44^
6. Transparency: solidarity depends on transparency, where claims that bind people together against a common “enemy,” the virus, can be scrutinized and believed. There are a whole series of disputable, misleading, and deceptive claims made that have undermined confidence in solidarity in the context of combatting COVID-19 in the United Kingdom. On testing: The health secretary Matt Hancock’s target of delivering 100,000 tests by the end of April was either side-stepped or subsequently not met in early May. On procurement, 1 billion pieces of PPE were miscounted (e.g., pairs of gloves were counted twice) and/or miscategorized (e.g., as disinfectant). Moreover, at the height of the PPE crisis, the protective standards of PPE were down-graded around the same time as COVID-19 was removed from the High Consequence Infectious Diseases list.^45^ On solidarity in public confidence in the lock-down: high-ranking Government officials like Dominic Cummings who clearly broke the spirit of lock-down rules did not resign from office. The defence of Dominic Cummings by Prime Minister and Cabinet colleagues added to public outrage. Dominic Cummings’ unrepentance and insistence that he had not broken the rules was difficult to accept by large swathes of the public who had to make painful personal choices in similar circumstances. Many felt unfairly treated and probably less likely to abide by the rules in the future.^46^

Having looked at human solidarity in the time of COVID-19, it is helpful to open up the discussion to understand solidarity less narrowly.

## Biocentric Solidarity in the Context of COVID-19

George Lakoff and Mark Johnson argue that the lives we lead are significantly influenced by the central metaphors we use.^47^ Given that the predominant metaphor around COVID-19 is about forging human solidarity to defeat a viral “enemy” it begs a deeper question about whether the virus is, indeed, our “enemy.”

Is this a good metaphor? Human enemies are usually living and malign. Yet it is arguable whether viruses are alive, let alone intentionally malign. Despite the obvious dis-analogy, it may still be a useful metaphor, given that CoV-SARS-2 infectivity rate leads to a highly disproportionate number of deaths from pneumonia-like symptoms brought on by COVID-19 in its most dangerous form. The virus as “the enemy” is really about human projection: we interpret the virus as we are, anything that kills indiscriminately “good” people and in such large numbers is like “an enemy” to us.

From a cooler and broader perspective, there is evidence to show that coronaviruses are not “enemies” to human kind in normal circumstances. Coronaviruses live naturally in nonhuman animal hosts like bats where they develop “super immunity” from the virus to prevent them from getting sick.^48^

Bats have been shown to carry 3,200 strains of coronavirus, the vast majority of them harmless, bar two closely related sarbevirus—one responsible for the SARS outbreak and the other COVID-19. Natural habitat loss caused by human-disturbed eco-systems leads to wild animals migrating to smaller and smaller areas where they end up in spaces that are more suited to human habitation. Close habitation of bats with humans increases the danger of zoonotic transfer through an intermediary species to humans.^49^

Roger Frutos et al. (2020) argues that the key to containing future epidemics is not to see the wild as an enemy, but to recognize that “human activities are responsible for the emergence and propagation of the zoonosis.” Habitat destruction leads to more contact of wild species that carry viruses that might be dangerous to humans, either because they live in closer proximity than normal, or wild animals are more available to humans for trade through unhygienic wet markets. In either case, the chance of zoonotic transfer and disease increases.^50^

It is, therefore, important to distinguish a shallower human-centered solidarity from a deeper biocentric solidarity. As we can appreciate an act of human-centered solidarity to tackle an emergency situation in a pandemic is necessary to save life and protect livelihoods in the short-term. However, an anthropocentric solidarity that also returns to business-as-usual in socioeconomic long-term looks complacent, when it is likely that our profligate way of life is likely to have caused the pandemic.

What we need is a bio-centric notion of solidarity in the longer term. Fritz Jahr argued for this years ago, recognizing that “in order to truly practice bioethics, one must be in solidarity with all forms of life.”^51^

Oblivious to the consequences of not practicing solidarity in this deeper sense before the pandemic, a reminder of Jahr’s call for biocentric solidarity is timely only if it is enacted in some way by a socioeconomic way of life that is sympathetic to it. This may be realizable if we live within the earth’s means to support diverse species on a healthy planet. This is, at least, still possible through enacting the new doughnut model of economics.^52^

## Notes

2.

Callaway E , Cyranoski D , Mallapay S , Sloye & Tollefson J . The coronavirus pandemic in five powerful charts. Nature 2020;579:482–3; available at https://www.nature.com/articles/d45186-020-00758-2 (last accessed 10 June 2020).3220336610.1038/d41586-020-00758-2

3. Solidarity. In Merriam-Webster.com; available at https://www.merriam-webster.com/dictionary/solidarity (last accessed 10 June 2020).

4.

Prainsack B , Buyx A . Solidarity in Medicine and Beyond. Cambridge: Cambridge University Press; 2017.

5. See note 4, Prainsack, Buyx 2017, at 4.

6. See note 4, Prainsack, Buyx 2017, at 42.

7.

Hadot P . Philosophy as a Way of Life. Davidson A, trans. Massachusetts: Blackwell; 1995.

8. See note 4, Prainsack, Buyx 2017, at 57–8.

9. Simelane H. COVID-19 and the call for solidarity: challenges for informal settlements. *Mail & Guardian*; 2020 April 12; available at https://mg.co.za/article/2020-04-12-covid-19-and-the-call-for-solidarity-challenges-for-informal-settlements/ (last accessed 10 Jun 2020).

10. Coronavirus: we are at war – Macron. *BBC News*; 2020 March 16; available at https://www.bbc.co.uk/news/av/51917380/coronavirus-we-are-at-war-macronMacron (last accessed 5 Jun 2020).

11. Boris Johnson’s address to the nation in full. *The Guardian*; 2020 Mar 23; available at https://www.theguardian.com/uk-news/2020/mar/23/boris-johnsons-address-to-the-nation-in-full (last accessed 10 Jun 2020).

12.

More T . More’s Utopia. Baker-Smith D, trans. Canada: University of Toronto Press; 2000.

13.

Walker P , Whittaker C , Watson O , Baguelin M , Ainslie K , Sangeeta B et al. The global impact of COVID-19 and strategies for mitigation and suppression. *Imperial College COVID-19 Response Team*; 2020 March 6; available at https://www.imperial.ac.uk/media/imperial-college/medicine/sph/ide/gida-fellowships/Imperial-College-COVID19-Global-Impact-26-03-2020v2.pdf (last accessed 10 Jun 2020).

14. See note 13, Ghani, Ferguson et al. 2020, at 2.

15. What counts as essential travel during the Covid-19 lockdown? *The UK’s Independent Fact Checking Charity*; 2020 June 5; available at https://fullfact.org/health/coronavirus-essential-travel/ (last accessed 10 Jun 2020).

16. See note 4, Prainsack, Buyx 2017, at 58.

17. Stay at home, protect the NHS, save lives. *Conservatives*; 2020 March 23; available at https://www.conservatives.com/news/stay-at-home-protect-the-nhs-save-lives (last accessed 10 Jun 2020).

18. A round of applause around the country. *#clapforourcarers*; 2020 March 25; available at https://clapforourcarers.co.uk/ (last accessed 10 Jun 2020).

19. Support for those affected by COVID-19, *GOV.UK*; 2020 April 9; available at https://www.gov.uk/government/publications/support-for-those-affected-by-covid-19/support-for-those-affected-by-covid-19 (last accessed 10 Jun 2020).

20. Varma T. Covid-19 and European solidarity: The fight for who we are. *European Council on Foreign Relations*; 2020 March 30; available at https://www.ecfr.eu/article/commentary_covid_19_european_solidarity_and_the_fight_for_who_we_are (last accessed 10 Jun 2020).

21. See note 20, Varma 2020.

22. Solidarity: Clinical trial for COVID-19 treatments. *World Health Organisation*; 2020 March 18; available at https://www.who.int/emergencies/diseases/novel-coronavirus-2019/global-research-on-novel-coronavirus-2019-ncov/solidarity-clinical-trial-for-covid-19-treatments (last accessed 10 Jun 2020).

23. See note 22, WHO 2020.

24. Kearney C. Italians sing patriotic songs from their balconies during coronavirus lockdown. *The Guardian*; 2020 March 14; available at https://www.theguardian.com/world/2020/mar/14/italians-sing-patriotic-songs-from-their-balconies-during-coronavirus-lockdown (last accessed 10 Jun 2020).

25. Trueman C. Pandemic leads to surge in video conferencing app downloads. *Computerworld*; 2020 April 3; available at https://www.computerworld.com/article/3535800/pandemic-leads-to-surge-in-video-conferencing-app-downloads.html (last accessed 10 Jun 2020).

26. Cottage industry of volunteers making face masks to protect NHS staff. *The Lochside Press*; 2020 March 28; available at https://thelochsidepress.com/2020/03/28/cottage-industry-of-volunteers-making-face-masks-to-protect-nhs-staff/ (last accessed 10 Jun 2020).

27. Zolfagharifard E. Meet the neighbours using WhatsApp groups to help the isolated elderly. *The Telegraph*; 2020 March 19; available at https://www.telegraph.co.uk/technology/2020/03/19/meet-neighbours-using-whatsapp-groups-help-isolated-elderly/ (last accessed 10 Jun 2020).

28. NHS responders scheme *NHS*; 2020 March 29; available at https://www.goodsamapp.org/NHS (last accessed 10 Jun 2020).

29. See note 28, NHS 2020.

30.

Foucault M . Of other spaces. Diacritics 1986;16(1):22–7. [Miskoweic J, trans.]

31.

Hetherington K . Badlands of Modernity: Heterotopia and Social Ordering. London and New York: Routledge; 1997.

32. See note 9, Simelane 2020.

33. Pidd H, Barr C, Mohdin A. UK’s corona divide. *The Guardian* 2020, May 5.

34. See note 2, Callaway, Cyranoski 2020.

35. See note 33, Pidd, Mohdin 2020, at 1.

36. Search results for care homes. *Channel 4 News*; 2020 May 18, 20, 21; available at https://www.channel4.com/news/search/care%20homes (last accessed 10 Jun 2020).

37.

Foucault M in Faubion JD . ed. Aesthetics, Method and Epistemology, Essential Works of Foucault, 1954–1984. Hurley R, trans. New York: New Press; 1998, at 175–85.

38. The UK’s official death toll for COVID-19 is the highest in Europe.

39. Freedman L. The real reason the UK government pursued herd immunity. *The New Statesman*; 2020 April 1; available at https://www.newstatesman.com/politics/uk/2020/04/real-reason-uk-government-pursued-herd-immunity-and-why-it-was-abandoned (last accessed 10 Jun 2020).

40. Mills J. UK death toll could have been halved if lockdown started week earlier. *The Metro*; 2020 June 10; available at https://metro.co.uk/2020/06/10/uk-death-toll-have-halved-lockdown-one-week-earlier-12833698/ (last accessed 11 Jun 2020).

41. Costello A. A public inquiry into the UK’s coronavirus response would find a litany of failures. *The Guardian*; 2020 April 1; available at https://www.theguardian.com/commentisfree/2020/apr/01/public-inquiry-coronavirus-mass-testing-pandemic (last accessed 10 Jun 2020).

42. Davies H. Private firm running UK PPE stockpile was sold in the middle of the pandemic. *The Guardian*; 2020 April 22; available at https://www.theguardian.com/world/2020/apr/22/revealed-private-firm-running-uk-ppe-stockpile-was-sold-in-middle-of-pandemic (last accessed 10 Jun 2020).

43. Brittan B. COVID-19: Joint procurement of PPE, ventilators and testing kits – What’s this about? 2020 April 24; available at http://www.bevanbrittan.com/insights/articles/2020/covid-19-eu-joint-procurement-of-ppe-ventilators-and-testing-kits/ (last accessed 10 Jun 2020).

44. See note 41, Costello 2020, at 13.

45. BBC statement on Panorama, Monday 27 April: Has government failed the NHS? *BBC Media Centre*; available at http://www.bbc.com/corporate2/mediacentre/statements/panorama-mon-27-apr (last accessed 10 Jun 2020).

46. Weaver M. Pressure on dominic cummings to quit over lockdown. *The Guardian*; 2020 May 22; available at https://amp.theguardian.com/politics/2020/may/22/dominic-cummings-durham-trip-coronavirus-lockdown (last accessed 10 Jun 2020).

47.

Lakoff G , Johnson M . The Metaphors We Live By. Chicago: University of Chicago Press; 1980.

48. Bat super immunity may explain how bats carry coronaviruses, study finds. *ScienceDaily*; 2020 May 6; available at https://www.sciencedaily.com/releases/2020/05/200506133614.htm (last accessed 10 Jun 2020).

49. Watts J. Promiscuous treatment of nature will lead to more pandemics – Scientists. *The Guardian*; 2020 May 7; available at https://www.theguardian.com/environment/2020/may/07/promiscuous-treatment-of-nature-will-lead-to-more-pandemics-scientists?CMP=Share_iOSApp_Other (last accessed 10 Jun 2020).

50.

Frutos R , Lopez Roig M et al. COVID-19: The conjunction of events leading to the coronavirus pandemic and lessons to learn for future threats. Frontiers of Medicine, Opinion Article 2020;12(5); available at https://www.frontiersin.org/articles/10.3389/fmed.2020.00223/full (last accessed 15 Jun 2020).10.3389/fmed.2020.00223PMC723541232574324

51.

Sass H-M . Fritz Jahr’s 1927 concept of bioethics. Kennedy Institute of Ethics Journal 2007;17(4):279–95.1836326710.1353/ken.2008.0006

52.

Raworth K . Doughnut Economics: Seven Ways to Think Like a 21-st Century Economist. St. Ives: Random House; 2017.

